# Severity of dry eye syndrome is related to anti-dsDNA autoantibody in systemic lupus erythematosus patients without secondary Sjogren syndrome: A cross-sectional analysis

**DOI:** 10.1097/MD.0000000000004218

**Published:** 2016-07-18

**Authors:** Alexander Chen, Hung-Ta Chen, Yih-Hsiou Hwang, Yi-Tsun Chen, Ching-Hsi Hsiao, Hung-Chi Chen

**Affiliations:** a Department of Medicine, Chang Gung University College of Medicine, Taoyuan; b Department of Ophthalmology, Chang Gung Memorial Hospital, Linkou; c Department of Internal Medicine, Taipei City Hospital-Heping Branch; d Department of Family Medicine, Taipei Veterans General Hospital, Taipei; e Center for Tissue Engineering, Chang Gung Memorial Hospital, Linkou, Taiwan.

**Keywords:** Anti-DNA antibodies, autoantibodies, complement proteins, secondary Sjogren syndrome

## Abstract

There are as many as one-third of the systemic lupus erythematosus (SLE) patients who suffer from dry eye syndrome. To this date, dry eye syndrome in SLE patients is believed to be caused by secondary Sjogren syndrome (sSS). However, there is increasing evidence for possible independency of dry eye syndrome and sSS in patients suffering from autoimmune diseases. The purpose of this retrospective observational case series was to identify SLE patients without sSS who had dry eye syndrome, examine the correlation of different autoantibodies and dry eye severity, and determine the cause of dry eye in these patients.

We included 49 consecutive SLE patients with dry eye who visited our dry eye clinic. In order to rule out sSS, these patients were all negative for anti-Sjogren's-syndrome-related antigen A and B (anti-SSA/SSB) and had no oral symptoms. Each patient's lupus activity was determined by serological tests including antidouble-stranded DNA antibody (anti-dsDNA), complement levels (C3, C4), erythrocyte sedimentation rate (ESR), and antinuclear antibody (ANA). Severity of dry eye syndrome was determined by corneal sensation (KSen), superficial punctuate keratopathy (SPK), Schirmer-I test (Schirmer), and tear film break-up time (TBUT). The autoantibodies and the dry eye parameters in each group were tested using the χ^2^ test or the Mann–Whitney *U* test for normally distributed or skewed data, respectively.

The anti-dsDNA showed significant correlations with KSen (*P* < 0.001), SPK (*P* < 0.001), and Schirmer (*P* = 0.042) but not TBUT. The C3 showed significant correlations with KSen (*P* < 0.001), SPK (*P* < 0.001), and Schirmer (*P* = 0.014) but not TBUT. No correlations of dry eye parameters were observed between C4, ESR, and ANA.

The major finding of this study was that the severity of dry eye syndrome in SLE patients without sSS was strongly correlated with anti-dsDNA and C3 but not with C4, ESR, and ANA.

## 1 Introduction

The morbidities of systemic lupus erythematosus (SLE), a systemic autoimmune disease, have been known to severely affect the life qualities of SLE patients.^[1,2]^ When predicting lupus's flare and assessing its activity with serological tests, elevation of antidouble-strand DNA antibody (anti-dsDNA) level^[3]^ and depression in complement levels (C3 and C4) are commonly used.^[4,5]^ Some studies suggest that the elevation of erythrocyte sedimentation rate (ESR) may also be effective for monitoring lupus activity.^[6]^ In addition, antinuclear antibody (ANA) is almost always tested in a patient who is suspected of having SLE. Despite the high diagnostic sensitivity of >95% for SLE,^[7]^ ANA has a relatively low specificity.^[8–10]^ Nonetheless, as anti-dsDNA, C3, C4, ESR, and ANA are all potentially related to lupus activity, we used these antibodies to correlate with the severity of dry eye in SLE patients.

Among the many morbidities of SLE, dry eye syndrome, also known as keratoconjunctivitis sicca that was first described by Henrik Sjogren in 1933, causes great distresses in approximately one-third of the SLE patients.^[11,12]^ Dry eye syndrome is also the most common ocular manifestation of SLE.^[11,12]^ Common tools for surveying dry eye syndrome are corneal sensation testing (KSen),^[13]^ superficial punctate keratopathy grading (SPK),^[14]^ Schirmer-I test (Schirmer), and tear film break-up time (TBUT),^[15–17]^ whereas common serum markers that are associated with dry eye syndrome are anti-Sjogren's-syndrome-related antigen A and B (anti-SSA and anti-SSB) and ESR.^[18]^ The reason that SLE-related dry eye syndrome studies included anti-SSA and anti-SSB was that SLE patients with dry eye syndrome were believed to be the cause of secondary Sjogren syndrome (sSS).^[19–22]^

However, recent studies have argued that dry eye syndrome may not be as closely associated with sSS as with SLE itself.^[23–25]^ Gilboe et al^[18]^ compared SLE patients with and without sSS and found that sicca symptoms (both oral and ocular) were common in SLE patients, but only few SLE patients were with sSS. Similarly, Fujita et al^[26]^ compared rheumatoid arthritis patients with and without sSS and found that 92% and 90% of the patients, respectively, developed dry eye, implying that the cause of dry eye cannot be completely attributed to sSS.

These findings suggested possible independency of dry eye syndrome and sSS as well as the uncertainty of the correlation between dry eye syndrome and SLE itself. Thus, we ruled out sSS by including only SLE patients with negative anti-SSA/SSB and no oral symptoms. We then analyzed and identified the correlation of dry eye severities and the titers of autoantibodies in these patients.

## 2 Patients and methods

### 2.1 Subjects

In an interval of 3 years, a total of 49 (45 enrolled, 4 excluded because serologic tests were 1 month apart from ophthalmic examinations) consecutive patients with SLE visited our dry eye clinic in Chang Gung Memorial Hospital and were carefully evaluated by 1 ophthalmologist (Chen HC). Diagnosis of SLE was made in the Rheumatology Clinic before the patients visited our dry eye clinic, according to criteria established by the Systemic Lupus International Collaborating Clinics (SLICC), an international group dedicated to systemic lupus erythematosus (SLE) clinical research, of which the classification criteria for SLE are ≧ 4 criteria (at least 1 clinical and 1 laboratory criteria) or biopsy-proven lupus nephritis with positive ANA or anti-dsDNA.^[1]^ Secondary Sjogren syndrome (sSS) was ruled out with the American-European Consensus Group (AECG) criteria (presence of sicca symptoms in addition to 2 objective tests for ocular and oral symptoms. The patients were all negative for anti-SSA/SSB and denied of having any oral symptoms. Despite the absence of oral symptoms, sSS could not be completely ruled out as objective tests were not performed. This retrospective cross-sectional study by chart review was approved by Institutional Review Board of Chang Gung Memorial Hospital, Linkou, Taiwan (Registration Number: 104-8842B).

### 2.2 Serologic tests

All laboratory data were obtained with standard laboratory procedures using 7600-210 Clinical Analayzer (Hitachi, Tokyo, Japan). Lupus activity was monitored serologically by levels of anti-double-strand DNA antibody (anti-dsDNA, IU/mL), complement C3 and C4 (mg/dL), and antinuclear antibody (ANA). Serum biochemical studies comprised erythrocyte sedimentation rate (ESR, mm), serum creatinine (Cr, mg/dL), and C-reactive protein (Hs-CRP, mg/mL).

### 2.3 Ophthalmic examinations

Although this was a retrospective study, routine ophthalmic examinations, including visual acuity, pneumotonometry, and slit-lamp biomicroscopy, were documented.

The standard for measuring corneal sensitivity was the Cochet–Bonnet esthesiometer (Luneau Ophthalmologia, Chartes Cedex, France), as previously described. This esthesiometer had a 60-mm-long nylon monofilament that can be adjusted in length. The filament was soft when fully extended and became firm when retracted into the handpiece, creating a pressure gradient that ranged from 11 to 200 mg/mm^2^. To measure corneal sensation, the nylon monofilament was applied smoothly and perpendicularly toward the corneal surface, avoiding touching the eyelashes, and contact was detected by the slightest bend of the nylon. If a participant did not note touch at the 6-cm length, the monofilament was downward adjusted at intervals of 5 mm until sensation was perceived. Patient reliability was tested by bringing the filament close to the cornea without actually touching. The length was recorded in millimeters (mm). Measurements were taken from the lesion location of the cornea with RCES, and the corresponding “image point” of the fellow healthy cornea. For example, if the primary lesion location was inferonasal OD, then the corresponding point would also be inferonasal OS.

In our dry eye clinic, tear function tests and ocular surface staining were also recorded. Tear function tests consisted of tear film break-up time (TBUT) and Schirmer-I test (Schirmer). For TBUT, applied before the corneal sensitivity test, a strip of moistened fluorescein paper (Haag-Streit, Konitz, Switzerland) was used to touch the inferior fornix for a short time with minimal stimulation. The tear film was observed under cobalt-blue-filtered light. The interval (seconds) between the last complete blink and the first emergence of randomly distributed dry spots was averaged from triplicate measurements. This was followed by staining with 1% Rose-Bengal solution. Both fluorescein and Rose Bengal staining scores were recorded and ranged between 0 and 9 points. For estimation of tear production, Schirmer was performed using standardized strips of filter paper (Alcon Laboratory, Fort Worth, TX), which were placed in the lateral canthus away from the cornea and left in place for 5 minutes with the eyes closed. Readings were recorded in millimeters of wetting for 5 minutes (mm /5 min).

### 2.4 Statistical analysis

Continuous variables were expressed as means ± standard deviation or frequencies. Student's *t* test was used to compare the means of continuous variables. Dry eye parameters and the presence of different autoantibodies in each group were tested using the χ^2^ test or the Mann–Whitney *U* test for normally distributed or skewed data respectively. Normality of the data was tested using Spearman's correlation test. Analyses were performed using SPSS for Window version 12.0 (SPSS, Inc., Chicago, IL). Statistical significance was defined as *P* < 0.05.

## 3 Results

A total of 49 SLE patients with dry eye syndrome were included in an interval of 3 years based on our inclusion criteria. Four were excluded because serologic tests were 1 month apart from ophthalmic examinations, leaving a total of 45 patients. The baseline clinical characteristics of these patients are listed in Table 1. All subjects (100%) were <50-year-old with a mean of 35.4, and 42 subjects (93%) were female. All subjects were anti-SSA/SSB negative without any oral symptoms. Complement C3 and C4 were both in the lower end of the normal range.

**Table 1:**
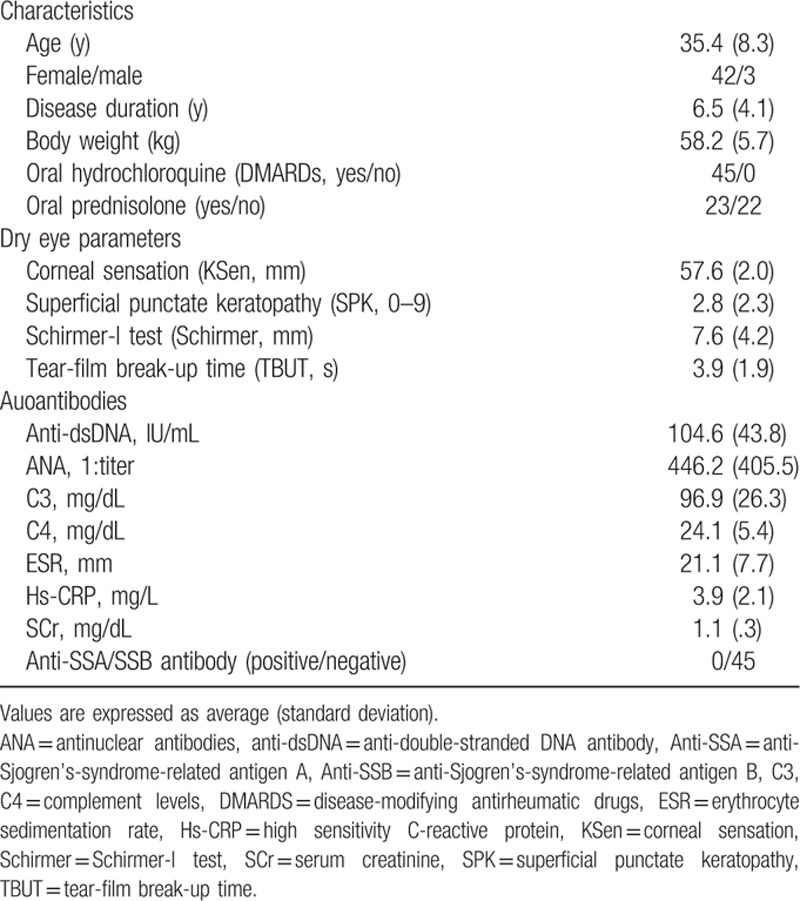
Profile of systemic lupus erythematosus patients who visited our dry eye clinic (N = 45).

The correlation between titers of the autoantibodies and the parameters of the dry eye tests is summarized in Table 2. The anti-dsDNA showed significant correlations with KSen (*P* < 0.001), SPK (*P* < 0.001), and Schirmer (*P* = 0.042) but not TBUT. The C3 showed significant correlations with KSen (*P* < 0.001), SPK (*P* < 0.001), and Schirmer (*P* = 0.014) but not TBUT. No correlations of dry eye parameters were observed between C4, ESR, and ANA.

**Table 2:**
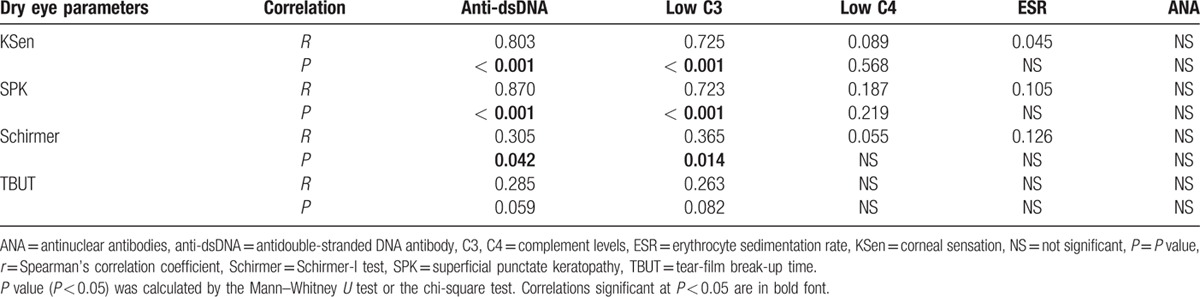
Spearman's rank correlation between titers of autoantibodies and dry eye parameters.

## 4 Discussion

**Figure 1:**
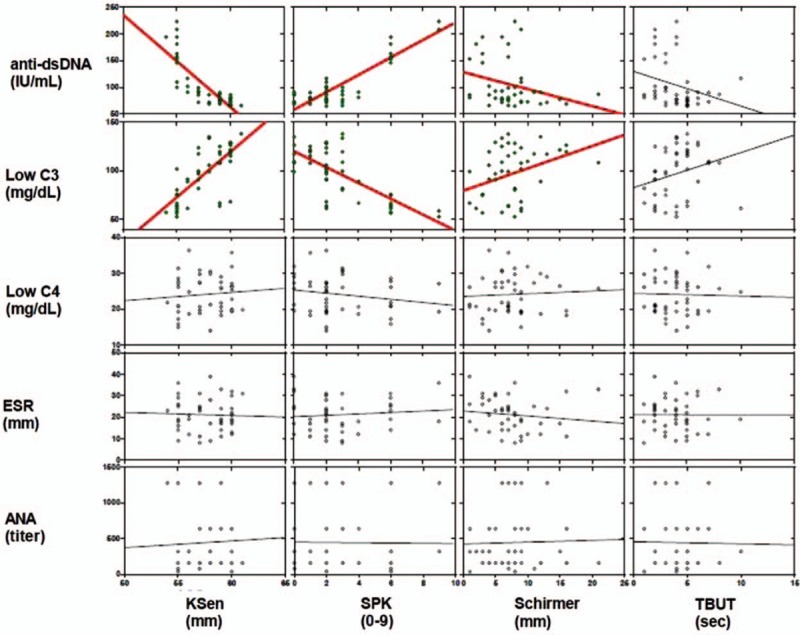
Spearman's rank correlation between titers of autoantibodies and dry eye parameters. The anti-dsDNA and C3 showed significant correlations with KSen, SPK, and Schirmer. No correlations of dry eye parameters were observed between C4, ESR, and ANA. ANA = antinuclear antibodies; anti-dsDNA = antidouble-stranded DNA antibody; C3, C4 = complement levels; ESR = erythrocyte sedimentation rate; KSen = corneal sensation; NS = not significant; *P* = *P*-value; *r* = Spearman's correlation coefficient; Schirmer = Schirmer-I test; SPK = superficial punctate keratopathy; TBUT = tear-film break-up time. ^∗^RGB.

Due to the increased evidences that dry eye syndrome in SLE patients may not be as much associated with sSS,^[18,23,25]^ we investigated the SLE patients who were anti-SSA/SSB negative without any oral symptoms to rule out sSS. The major finding of this study was that the severity of dry eye syndrome in these patients was strongly correlated with anti-dsDNA and C3 but not with C4, ESR, and ANA (Fig. 1).

Anti-dsDNA is regarded as highly specific for SLE because it has strong positive correlation with the commorbidities of SLE,^[3–5,27–29]^ but its association with dry eye syndrome has not been clearly identified. Our study found that anti-dsDNA was significantly correlated with KSen (*P* < 0.001), SPK (*P* < 0.001), and Schirmer (*P* = 0.042). Interestingly, Menendez et al^[30]^ found that SLE patients with positive anti-SSA had lower levels of anti-dsDNA, but the results were not statistically significant. To the best of our knowledge, our study was the first to show that SLE patients with dry eye syndrome and negative anti-SSA were associated with high levels of anti-dsDNA. And as anti-dsDNA is rarely found in other autoimmune diseases,^[31]^ our result offered a new perspective on the cause of dry eye. Previous studies have already shown that anti-dsDNA was effective in monitoring lupus activity and that the rise of anti-dsDNA level could predict SLE relapse.^[4,5]^ However, the role of anti-dsDNA on the pathogenesis of dry eye in SLE, or even kidney injury, remains to be elucidated. Likewise, the pathogenesis of dry eye in Sjogren syndrome is still ambiguous, but the targeting of glandular epithelial cells of the lacrimal glands by lymphocytes is thought to be one of the causes.^[32]^ Yung et al^[33]^ recently revealed that anti-dsDNA induced a series of proinflammatory cytokines such as TNF-α and interleukins in proximal renal tubular epithelial cells, which led to kidney inflammation in SLE. Consequently, we speculate that anti-dsDNA also plays a pivotal role in lymphocyte infiltration of the lacrimal glands, causing destruction of the epithelial cells and thus the eye dryness. Nonetheless, these are all hypothesis that remains for future investigations.

Some studies have shown that low complement levels (C3 and C4) are often associated with many comorbidities of SLE.^[4,5]^ Our study indicated that only C3 but not C4 was significantly correlated with dry eye syndrome in terms of KSen (*P* < 0.001), SPK (*P* < 0.001), and Schirmer (*P* = 0.014). Kao et al^[34]^ have shown that C3 was better than C4 in evaluating lupus activity because C4 was not involved in the alternative complement pathway. Lloyd and Schur^[35]^ also showed that C4 often remained low despite remission of lupus activity. Moreover, Vasilev et al^[36]^ illustrated that C3 played a crucial role in disrupting the alternative complement pathway in patients with lupus nephritis. On the contrary, in a recent review by Papagiannuli et al,^[21]^ C4 was pointed out to be particularly important for the clearance of immune complexes in SLE via the classical complement pathway. In other words, low C3 and C4 are both important indicators for lupus activity due to their involvements in classical and alternative complement pathways, but C4 is less capable of monitoring lupus activity. The above can partially explain our finding that C3, but not C4, was strongly correlated with dry eye severity in SLE patients. Nonetheless, it is worth mentioning that Menendez et al^[30]^ found lower complement levels to be positively associated with positive anti-SSA, but only levels of C4, not C3, were statistically significant. Our findings agreed with the implication of Menendez et al because our patients were all anti-SSA negative and showed lower complement levels, but only statistically significant for C3. Based on Menendez et al's results and our findings, we can infer that the level of C3 and C4 are somehow dependent on the presence of anti-SSA. Also, the cause of dry eye syndrome in SLE patient without anti-SSA may be more attributed to C3 as to C4.

There has been no consensus on the association of ESR and lupus activity thus far. In a prospective study by Mirzayan et al,^[3]^ high levels of ESR have been shown to correlate with the frequency but not the severity of flare. On the contrary, a recent study by Stojan et al^[6]^ reported ESR as an effective tool for assessing lupus activity, suggesting all levels of ESR to be strongly correlated with disease activity. The author did suggest, however, that the significance may have been overestimated, as the extent of difference in the correlation of ESR and disease activity was minimal. In our study, there was no evidence of correlation between ESR and dry eye severity, entailing the lack of correlation with SLE. We suggest future study to include the analysis of ESR and dry eye severity in SLE patients to further confirm the correlation.

ANA is deemed extremely sensitive to SLE,^[1,3–5,28]^ but no correlation was observed between ANA and the dry eye parameters in our study. There are several reasons that may explain this lack of correlation. First of all, ANA is relatively common in healthy individuals and other autoimmune diseases.^[8–10]^ In addition, ANA is also significantly higher in females^[37]^ and non-SLE elderly patients.^[38]^ Moreover, patients who have undergone treatment or have had longstanding disease can lose the ANA reactivity, which is why ANA is rarely used to monitor lupus activity. The above reasons support the fact that ANA has very low specificity for SLE in spite of its high sensitivity. Hence, we were not surprised that ANA was not correlated with the severity of dry eye in our SLE patients.

Out of the four dry eye parameters used in this study, only TBUT showed no correlation with any of the autoantibodies. This finding was unexpected as TBUT has been commonly used for analysis in various types of dry eye diseases such as rheumatoid arthritis, Sjogren syndrome, and graft-versus-host disease, and so on. Evidences have shown that TBUT is not a good indicator for assessing dry eye severity due to its inaccuracy.^[39,40]^ This is also the reason that new methods like tear film break-up pattern, symptomatic break-up time, and ocular protection index are being developed to further improve the diagnostic value of TBUT.

There were some limitations in our study, mainly bounded by the fact that it was a retrospective study. First, even though our SLE patients denied having oral symptoms, we did not perform lip biopsy and sialography to completely rule out the possibility of sSS. Second, the sample size was relatively small, thereby increased the type II error in our study. Third, 92% (42/45) of the patients were females. The differences between female and male cannot possibly be distinguished under this circumstance. Fourth, selection bias existed, as the subjects all came from 1 dry eye clinic.

In summary, in SLE patients with negative anti-SSA/SSB, dry eye severity was strongly correlated with anti-dsDNA and C3 but not with C4, ESR, and ANA. The strong correlation of anti-dsDNA with dry eye severity may be due to the involvement of anti-dsDNA in the lymphocyte infiltration of the lacrimal glands, but further studies are needed. As anti-dsDNA is highly specific for SLE and is highly correlated with dry eye severity, our findings have established a strong evidence of association between dry eye severity and SLE patients with negative anti-SSA/SSB. Thus, we suggest that dry eye may be added to the criteria for SLE diagnosis.
